# Key Evolutionary Transformations in *Homo sapiens*

**DOI:** 10.4061/2011/478690

**Published:** 2012-03-08

**Authors:** Darren Curnoe, Bing Su, Parth Chauhan, John Gowlett

**Affiliations:** ^1^School of Biological, Earth and Environmental Sciences, University of New South Wales, Sydney, NSW 2052, Australia; ^2^State Key Laboratory of Genetic Resources and Evolution, Kunming Institute of Zoology and Kunming Primate Research Centre, Chinese Academy of Sciences, Kunming 650223, China; ^3^Stone Age Institute, 1392 W. Dittemore Road, Gosport, IN 47433, USA; ^4^School of Archaeology, Classics and Egyptology, 2-14 Abercromby Square, University of Liverpool, Liverpool L69 7WZ, UK

The origin of modern humans remains a central question of palaeoanthropology—the discipline devoted to the scientific investigation of human evolution. This research field, like the questions it asks and evidence it draws upon, is multidisciplinary and synthetic in nature. Palaeoanthropology is also in the mist of a transformation, from a once largely descriptive and discovery driven enterprise to a discipline integrating the methods and results of cutting edge discoveries in 3D modeling and morphometrics, genomic sequencing, including ancient DNA and geochronology and isotope geochemistry, among others. Thus, the unfolding story of recent human evolution is as much about the transformation of the history of human evolution research as it is about the evidence and narratives we weave about of our origins.

The geographic location and timing of the origin of modern humans are now well understood to have been Sub-Saharan Africa at around 200,000 years ago. Yet, there is still much to be learned about the hypothesised divergence of *Homo sapiens *and *H. neanderthalensis *from a common ancestor some 400,000 years ago, the deep-time transformations of archaic humans to modern *H. sapiens*, and the colonisation of Eurasia by a subset of Sub-Saharan Africans after 100,000 years ago.

Moreover, attempts to define anatomical and behavioural modernity have become increasingly challenging with new research in genetics suggesting the possibility of interbreeding with archaic hominins and discoveries from archaeology blurring the behavioural distinctions between Neanderthals and early modern humans.

Finally, a historical subtext in human origins research deals with explaining the perceived “gulf” between humans and all other life. Yet, this notion is one that sits uncomfortably beside the Darwinian concept of evolutionary continuity through common descent. It is also a subtext built into the very language of human origins research, being evident in widely used terms like “modern” and “archaic.” In reality, such terms lack biological meaning and are arbitrary (descriptive) contrivances—for example, if *H. sapiens *and *H. neanderthalensis* actually share a common ancestor, then the latter can be no more archaic than the former; yet *H. neanderthalensis* is frequently referred to as a perhaps the ultimate example of an archaic group. Such terms are clearly loaded and only muddle our attempts to reconstruct human biological history within an evolutionary framework.

This special issue takes a broad brush approach to examining some of the more important topics of contemporary palaeoanthropology. Contributions cover topics ranging from molecular to morphological and population level transformations, and some of the key factors underpinning human evolution and variability, through to important behavioural questions examined using isotopic proxies, ideas from evolutionary psychology, and rich data from the archaeological record. They should find wide relevance across the many disparate fields that comprise this enterprise of human evolutionary research.

We wish to express our sincerest appreciation to all of the authors and reviewers whose efforts have made this special issue such a success. We truly hope it will provide a sense of “where we're at” with respect to some key questions, answer some old questions, and, most importantly, satisfy curiosity and excite readers about future possibilities.

*Darren Curnoe* *Darren Curnoe* *Bing Su* *Bing Su* *Parth Chauhan* *Parth Chauhan* *John Gowlett* *John Gowlett*

